# Critical role of cellular communication network factor 1 in early osteogenesis of mesenchymal stem cells primed by low‐intensity pulsed ultrasound and bone morphogenetic protein 2

**DOI:** 10.1002/ccs3.70101

**Published:** 2026-08-26

**Authors:** Hsu Myat Paing, Satoshi Kubota, Masaharu Takigawa, Takuo Kuboki, Takashi Nishida

**Affiliations:** ^1^ Department of Biochemistry and Molecular Dentistry Okayama University Graduate School of Medicine Dentistry and Pharmaceutical Sciences Okayama Japan; ^2^ Department of Oral Rehabilitation and Regenerative Medicine Okayama University Graduate School of Medicine Dentistry and Pharmaceutical Sciences Okayama Japan; ^3^ Advanced Research Center for Oral and Craniofacial Sciences Okayama University Faculty of Medicine Dentistry and Pharmaceutical Sciences Okayama Japan

**Keywords:** BMP‐2, CCN1, low‐intensity pulsed ultrasound (LIPUS), mesenchymal stem cells, osteogenic differentiation

## Abstract

There is a known reciprocal inhibitory relationship between adipogenesis and osteogenesis in mesenchymal stem cells (MSCs). Our previous study showed that low‐intensity pulsed ultrasound (LIPUS) did not promote osteogenesis of a murine MSC line C3H10T1/2, although LIPUS treatment suppressed adipogenesis. Therefore, we investigated the effect of a combination of an osteoinductive factor such as bone morphogenic protein‐2 (BMP‐2) and LIPUS treatment on osteogenesis. This combination significantly upregulated the gene expressions of BMP receptors and RUNX2, which is a key early osteogenic transcription factor. Interestingly, cellular communication network factor 1 (CCN1) was significantly increased at the mRNA and protein levels. Additionally, *Runx2* expression was increased by a recombinant CCN1 protein and decreased in CRISPR‐Cas9‐generated *Ccn1* knockout (KO) cells treated with the combination of LIPUS treatment and BMP‐2. Moreover, *Runx2* expression was recovered through the transfection of a *Ccn1* expression plasmid into *Ccn1* KO cells. These findings indicate that treatment with the combination of LIPUS treatment and BMP‐2 promoted CCN1 production, and the CCN1 regulates the *Runx2* expression in C3H10T1/2 cells, thus suggesting that CCN1 induced by mechanical stimulation and an osteoinductive factor plays a pivotal role in osteogenesis of MSCs.

## Graphical abstract

Cooperative stimulation of mechanical stress and osteoinductive factor, such as BMP‐2, enhances BMP signaling via the upregulation of BMP receptors. As a result, CCN1 production is increased and is secreted to the extracellular environment. Secreted CCN1 promotes Runx2 expression through interaction with integrin, leading to early osteogenic differentiation of mesenchymal stem cells.

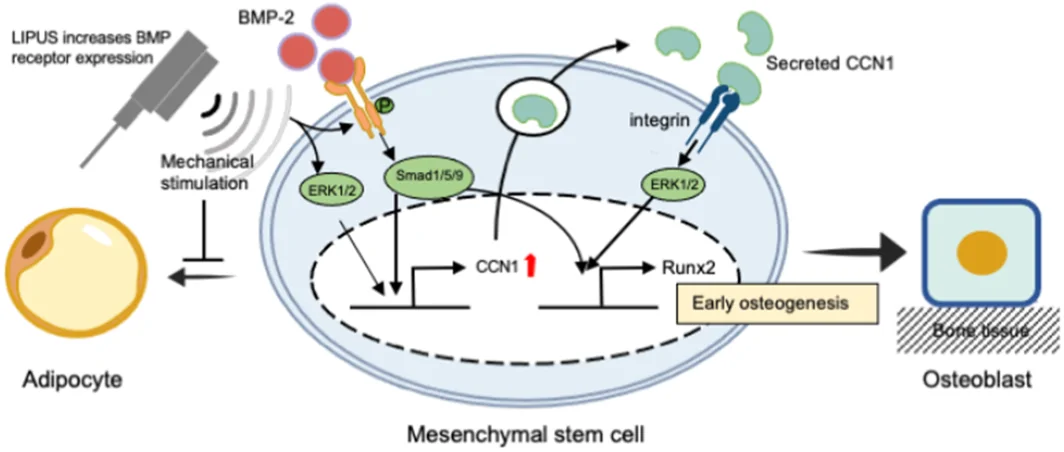

## 1 INTRODUCTION

Dental implants fixed into the jawbone as an artificial tooth root are among the therapeutic methods currently available for oral rehabilitation.^1^ The early achievement of direct binding between dental implant and alveolar bone, namely osseointegration, is important in making this treatment successful.^1^ Low‐intensity pulsed ultrasound (LIPUS) is a noninvasive mechanical stimulation that has been reported for its potential to treat bone fractures with nonunion and to promote soft tissue healing.^2^, ^3^, ^4^ Therefore, LIPUS has been used clinically to achieve implant stability in the jawbone^5^, ^6^; however, the cellular and molecular mechanisms by which LIPUS stimulates osseointegration remain unclear. It is well known that mesenchymal stem cells (MSCs) have the ability to self‐renew and differentiate into various cell types, such as osteoblasts, chondrocytes, adipocytes, fibroblasts, and myoblasts, and play a crucial role in tissue repair.^7^ The pluripotency of MSCs is characterized by the reciprocal relationship between adipogenesis and osteogenesis^8^, ^9^ or between adipogenesis and myogenesis,^10^ that is, a committed lineage is typically accompanied by suppression of another. This exclusive relationship is regulated by lineage‐specific transcription factors. For instance, adipogenic differentiation is mainly governed by CCAAT/enhancer‐binding protein‐α (C/EBPα) and peroxisome proliferator‐activated receptor‐γ (PPARγ).^11^ We previously demonstrated that adipogenesis was effectively suppressed by LIPUS stimulation alone in a murine MSC cell line, C3H10T1/2.^12^ However, despite the suppressive effect of LIPUS treatment on adipogenesis, it had no effect on the gene expression of osteogenic differentiation markers such as *Runx2*, *Sp7*, and *alkaline phosphatase* (*Alp*) (Supporting Information Figure S1). These findings suggest that although LIPUS can inhibit adipocyte lineage commitment, it is insufficient to determine the fate of MSCs toward osteoblasts. Interestingly, we showed that LIPUS treatment significantly increased the expression of cellular communication network factor 2 (CCN2)^12^ CCN2 is a matricellular protein, and it plays important roles in cell proliferation, chondrocytes^13^ and osteoblast development,^14^ wound healing,^15^ tissue regeneration,^16^ and so on. Additionally, CCN2 belongs to the CCN family of proteins, which is composed of six members.^17^ It has been reported that CCN family proteins have multifunctional biological effects.^17^ Among them, CCN1 and CCN2 are particularly known to regulate bone development via interaction with several growth factors, including fibroblast growth factors, transforming growth factor‐β, and bone morphogenetic protein‐2 (BMP‐2).^18^ Considering that BMP‐2 is one of the well‐established factors with proven clinical efficacy in bone regeneration, the combination of mechanical stimulation, such as LIPUS with BMP‐2, may efficiently promote osteogenic differentiation by controlling the behavior of CCN family proteins.

Based on our previous findings^12^ and this idea, we examined whether the differentiation of MSCs to osteoblasts is promoted by LIPUS treatment combined with an osteoinductive factor. Furthermore, we hypothesized that CCN family proteins induced by their combination are the main contributors to the osteogenesis. Hence, the aim of this study is to investigate whether or not LIPUS treatment combined with BMP‐2 enhances the BMP signaling pathway and induces CCN family proteins, which may be involved in the osteogenesis of MSCs as key mediators of osteoblast differentiation.

## 2 MATERIALS AND METHODS

### 2.1 Materials

Alpha‐modification of Eagle's medium (αMEM) and fetal bovine serum (FBS) were purchased from MP Biomedicals (Solon, OH, USA) and Gibco, Thermo Fisher Scientific, respectively. Plastic dishes and multiwell plates were obtained from Thermo Fisher Scientific. Recombinant BMP‐2 and CCN1 were obtained from Osteopharma Inc. and PeproTech, respectively. Anti‐CCN1 and anti‐CCN2 antibodies were purchased from Proteintech Group Inc.. Anti‐CCN4 antibody came from Santa Cruz Biotechnology Inc., and anti‐phospho‐Smad1/5/9, anti‐phospho‐ERK1/2, anti‐total ERK1/2 antibodies, and horseradish peroxidase (HRP)‐conjugated anti‐rabbit IgG and anti‐mouse IgG were obtained from Cell Signaling Technology. Anti‐β‐actin antibody and *β*‐glycerophosphate were obtained from MilliporeSigma. A list of catalog numbers of antibodies used in this study is provided in Supporting Information (Table S1). Ascorbic acid came from Nacalai Tesque, Inc.

### 2.2 Cell culture

The murine MSC line C3H10T1/2 was obtained from the RIKEN Cell Bank (RCB0247; RRID: CVCL_0190, Ibaraki, Japan) and this cell line was originally derived from mouse embryo (species: *Mus musculus*, sex unspecified, fibroblast‐like morphology). We confirm that this cell line was authenticated regarding use for basic research and was free of *mycoplasma* contamination for following experiments. C3H10T1/2 cells were inoculated at a density of 2 × 10^4^/cm^2^ into a culture dish and multiwell plate containing αMEM supplemented with 10% FBS and was cultured at 37°C in a humidified atmosphere with 5% CO_2_.^12^, ^19^

### 2.3 Osteogenic differentiation

For osteogenic differentiation, C3H10T1/2 cells were inoculated at a density of 2 × 10^4^ cells per 35 mm culture dish and were cultured until they reached sub‐confluence. The medium was then replaced with an osteogenic differentiation medium containing ascorbic acid (10 μg/mL) and *β*‐glycerophosphate (10 mM), and the cells were cultured for 4 days. Then, the medium was changed with fresh osteogenic differentiation medium, and the cells were cultured for an additional 3 days. For 3 days before the end of experiment, LIPUS was applied in the presence or absence of recombinant BMP‐2 at a final concentration of 100 ng/mL.

### 2.4 LIPUS treatment

In this study, an ST‐SONIC apparatus was provided from ITO Corporation Ltd..^12^, ^20^ Briefly, C3H10T1/2 cells were cultured in 35 mm culture dishes in αMEM containing 10% FBS until they reached sub‐confluence. Then, the medium was replaced with osteogenic differentiation medium, and the cells were cultured for 7 days. For 3 days before the end of the experiment, the cells were exposed to LIPUS at a frequency of 3.0 MHz and the intensity was 60 mW/cm^2^ for 20 min once a day.^12^, ^20^ During LIPUS treatment, the cells were maintained in a humidified atmosphere of 5% CO_2_ at 37°C. Control samples were maintained under the same conditions but without treatment with LIPUS.

### 2.5 Quantitative reverse‐transcription‐PCR analysis

Total RNA was extracted from C3H10T1/2 cells using the RNeasy Mini kit (Qiagen, Hilden, Germany) following the manufacturer's protocol. RNA concentration and purity were determined by spectrophotometrical analyzer (DU 730, Beckman Coulter, Brea, CA, USA), and first‐strand cDNA was synthesized using the PrimerScript^TM^ RT Reagent kit (Takara Bio, Shiga, Japan). Quantitative polymerase chain reaction (PCR) was subsequently performed with SYBR Green Real‐time PCR Master Mix (Toyobo, Osaka, Japan) and specific primers for target genes (Supporting Information Table S2A) using a StepOnePlus™ Real‐Time PCR System (Applied Biosystems). Glyceraldehyde 3‐dehydrogenase gene (*Gapdh*) was used as an internal control, and gene expression levels were evaluated using the 2^−ΔΔCt^ method.^21^

### 2.6 Western blot analysis

Whole‐cell lysates were collected by lysis buffer (20 mM Tris‐HCl pH 7.7, 150 mM NaCl, 1 mM EDTA, and 1% Triton X‐100) and were sonicated on ice. Then, cell lysate was centrifuged at 12,000 rpm for 5 min to remove debris. Protein concentrations of the cell lysates were quantified using a BCA protein assay kit (Thermo Fisher Scientific, USA) with serially diluted bovine serum albumin as a standard. A western blot analysis was performed as described previously.^22^ The cell lysate mixed with SDS sample buffer (0.5 M Tris‐HCl pH 6.8, 10% SDS, 20% glycerol, and 2% *β*‐mercaptoethanol) was separated using SDS–polyacrylamide gel electrophoresis (SDS‐PAGE), and then transferred onto polyvinylidene difluoride (PVDF) membranes (MilliporeSigma) using a semidry transfer apparatus (Atto corporation). Blots were then reacted overnight at 4°C with the primary antibodies indicated in Supporting Information Table S1. Subsequently, the membranes were washed with Tris‐buffered saline (TBS) with Tween 20 (TBST) and TBS buffers, and the blots were reacted at room temperature with the appropriate HRP‐conjugated secondary antibodies for 1 h. After washing with TBST and TBS buffers for 15 and 10 min, respectively, the bands were visualized and analyzed via chemiluminescence (LAS‐4000 imaging system, Fujifilm). The band intensities were quantified using ImageJ software, and signals were normalized to *β*‐actin as a loading control.

### 2.7 Generation of Ccn1‐knocked out cells using CRISPR‐Cas9 system

To generate *Ccn1*‐deficient C3H10T1/2 cells, genome editing was performed using a CRISPR–Cas9 system.^23^ Recombinant Cas9 protein (Guide‐it™, Takara Bio, Cat. #632641) and two guide RNAs (gRNAs) designed for the N‐terminal and C‐terminal sequences of the mouse *Ccn1* coding region were transfected into C3H10T1/2 cells by electroporation using a NEPA21 apparatus (NEPA Gene Co., Ltd.). The primer sequences used to synthesize the gRNAs are indicated in Supporting Information Table S2B. The gRNAs were synthesized using the GeneArt™ Precision gRNA Synthesis kit (Invitrogen, Thermo Fisher Scientific). To confirm transfection efficiency, pmaxGFP™ plasmid (Lonza Cologne GmbH) combined with the Cas9–gRNA complexes was transfected into the cells. Next day, single‐cell clones expressing GFP were selected through serial dilution, and the deletion of *Ccn1* in each clone was verified by genomic PCR analysis, followed by Sanger sequencing.

### 2.8 Genomic PCR analysis for genotyping

Genotypes were initially screened by PCR analysis using Quick Taq™ HS DyeMix (TOYOBO, Osaka, Japan) with specific primers indicated in Supporting Information Table S2C. PCR products were analyzed by agarose gel electrophoresis to detect deletion events. Loss of CCN1 protein production was further confirmed by western blot analysis.

### 2.9 Transfection with the Ccn1 expression plasmid

The full‐length coding region of mouse *Ccn1* was amplified with the PCR technique using cDNA derived from NIH3T3 fibroblasts as a template and specific primers indicated in Supporting Information Table S2D. The amplicon, bearing a 8xHis‐tag at the C‐terminal region, was inserted into a pFlag‐CMV (Invitrogen, Carlsbad, CA, USA), and the nucleotide sequence of this plasmid was confirmed using Sanger sequencing. *Ccn1*‐knocked out cells were transfected with either the *Ccn1* expression plasmid or a pFlag‐CMV empty vector (EV) by electroporation. A pmaxGFP™ plasmid (Lonza Cologne GmbH) was co‐transfected to confirm transfection efficiency. GFP‐positive cells were observed the following day using fluorescence microscopy to evaluate transfection efficiency (Supporting Information Figure S2). Total RNA and cell lysate were collected 48 h after transfection for subsequent analyses.

### 2.10 Statistical analysis

All experiments were conducted on at least two independent occasions, yielding consistent results. Data were analyzed for differences among multiple groups and between two groups using one‐way ANOVA followed by Tukey's HSD post hoc test, as well as an unpaired Student's *t*‐test, all of which were performed using SPSS version 25. Statistical significance was evaluated at *p* < 0.05. Graphical representations were generated with GraphPad Prism version 9.

## 3 RESULTS

### 3.1 Combination of LIPUS treatment and BMP‐2 mediates BMP signaling via upregulation of BMP receptors

**FIGURE 1:**
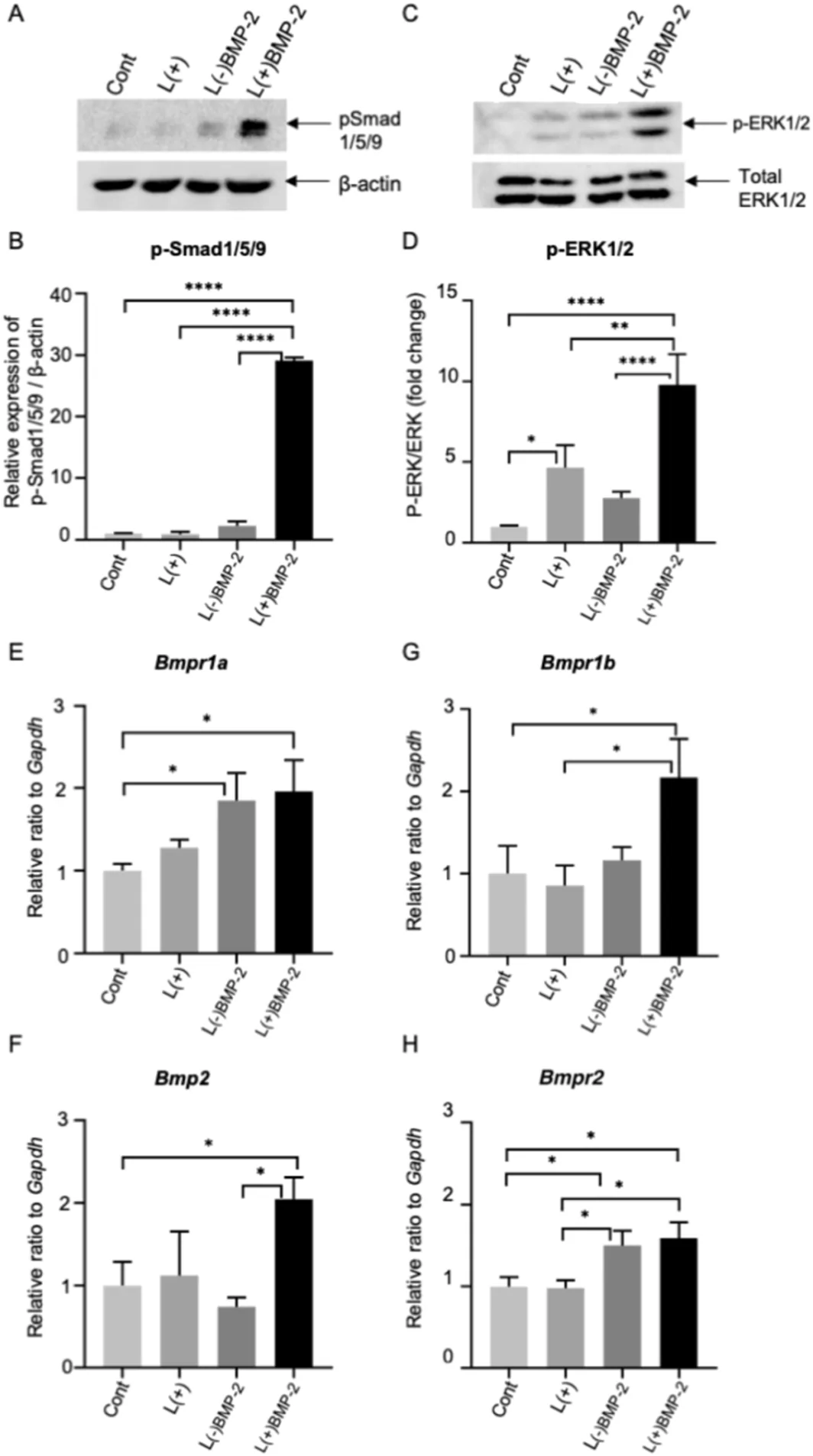
Activation of BMP signaling and the gene expressions of *Bmp‐2* and *Bmp receptors* in C3H10T1/2 cells by a combination of LIPUS and BMP‐2. (A) C3H10T1/2 cells were inoculated into 35‐mm diameter dishes and cultured in αMEM containing 10% fetal bovine serum. Next day, the medium was changed to osteogenic medium, and the cells were cultured until they reached sub‐confluence. Then, the cells were added to recombinant BMP‐2 at a concentration of 100 ng/mL and then immediately treated with LIPUS at a frequency of 3.0 MHz and an intensity of 60 mW/cm^2^. After 20 min of the treatment, the cell lysate was collected and western blot analysis was performed using anti‐phospho‐Smad1/5/9 (60 kDa) and anti‐β‐actin loading control antibodies. (B) The band intensities of phospho‐Smad1/5/9 and *β*‐actin were measured by ImageJ, and the band intensities of phospho‐Smad1/5/9 were normalized to those of *β*‐actin. Data showed the mean ± SD (*n* = 3). Statistical significance was calculated based on the relative density normalized to *β*‐actin (****p < 0.0001) (C) Representative western blot showing phosphorylation of ERK1/2 following treatment with LIPUS, BMP‐2, or the combined stimulation in C3H10T1/2 cells. Phospho‐ERK1/2 bands were detected at an apparent molecular weight of approximately 42–44 kDa. Total ERK1/2 was used as the internal control. (D) Quantification of phospho‐ERK1/2 normalized to total ERK1/2. Data are presented as mean ± SD (*n* = 5), and statistical significance was determined using one‐way ANOVA followed by Tukey's HSD post hoc test. (*p < 0.05, **p < 0.01, ***p < 0.001, and ****p < 0.0001). (E–H) Quantitative RT‐PCR analysis of the gene expressions of *Bmpr1a* (E), *Bmp‐2* (F), *Bmpr1b* (G), *and Bmpr2* (H). The gene expression levels were normalized to that of *Gapdh*. Statistical analysis was performed using one‐way ANOVA followed by Tukey's HSD post hoc test (*p < 0.05) (*n* = 6 per group); all experiments were performed in triplicate. Cont: without BMP‐2 and LIPUS treatment; L (+): LIPUS treatment alone; L (−)BMP‐2: BMP‐2 stimulation alone; and L (+) BMP‐2: both BMP‐2 and LIPUS treatment.

To determine whether or not LIPUS treatment mediates the phosphorylation of Smad1/5/9, which are main mediators for the canonical BMP signaling pathway, we first performed western blot analysis using the anti‐phospho‐Smad1/5/9 antibody. After C3H10T1/2 cells had reached sub‐confluence, they were treated with LIPUS with or without BMP‐2 at a concentration of 100 ng/mL for 20 min. As shown in Figure 1A, although the production of phospho‐Smad1/5/9 was not affected by LIPUS treatment without BMP‐2 in C3H10T1/2 cells, the production was remarkably increased by LIPUS treatment with BMP‐2. To quantify these data, the intensity of this band was measured using a densitometer. As shown in Figure 1B, the intensities of the band for phospho‐Smad1/5/9 were significantly increased by treatment with LIPUS combined with BMP‐2 compared with those with other stimulations. These findings indicate that LIPUS treatment combined with BMP‐2 synergistically enhances BMP signaling in MSCs. We then assessed ERK1/2 activation via a potential noncanonical BMP signaling pathway. As shown in Figure 1C, the phosphorylation of ERK1/2 was modestly increased by LIPUS treatment alone and further elevated by the combined LIPUS and BMP‐2 stimulation. Quantitative analysis demonstrated that phospho‐ERK1/2 levels were significantly increased in the combined treatment group (Figure 1D), whereas total ERK1/2 levels remained unchanged. Thus, in addition to Smad activation, the combined LIPUS and BMP‐2 stimulation significantly upregulated ERK1/2 phosphorylation, indicating activation of MAPK signaling pathways. Next, to investigate the mechanism for this synergistical effect, after the combination of LIPUS treatment and BMP‐2 was repeated three times, gene expressions of *Bmp2* and *Bmpr*s were examined for each treatment, namely, LIPUS treatment alone, BMP‐2 treatment alone, and their combination. As shown in Figure 1F,G, *Bmp2* and *Bmpr1b* expressions were the highest with combined LIPUS and BMP‐2 treatments and *Bmpr1a* and *Bmpr2* expressions were comparable to those found with BMP‐2 stimulation alone (Figure 1E,H). These results indicate that the combination of LIPUS treatment and BMP‐2 enhances the expressions of both *Bmp2* and *Bmpr*s, suggesting that upregulated expressions of their combination lead to synergistic control of the BMP signaling pathway.

### 3.2 Combination of LIPUS and BMP‐2 promotes early osteogenic gene expression

**FIGURE 2:**
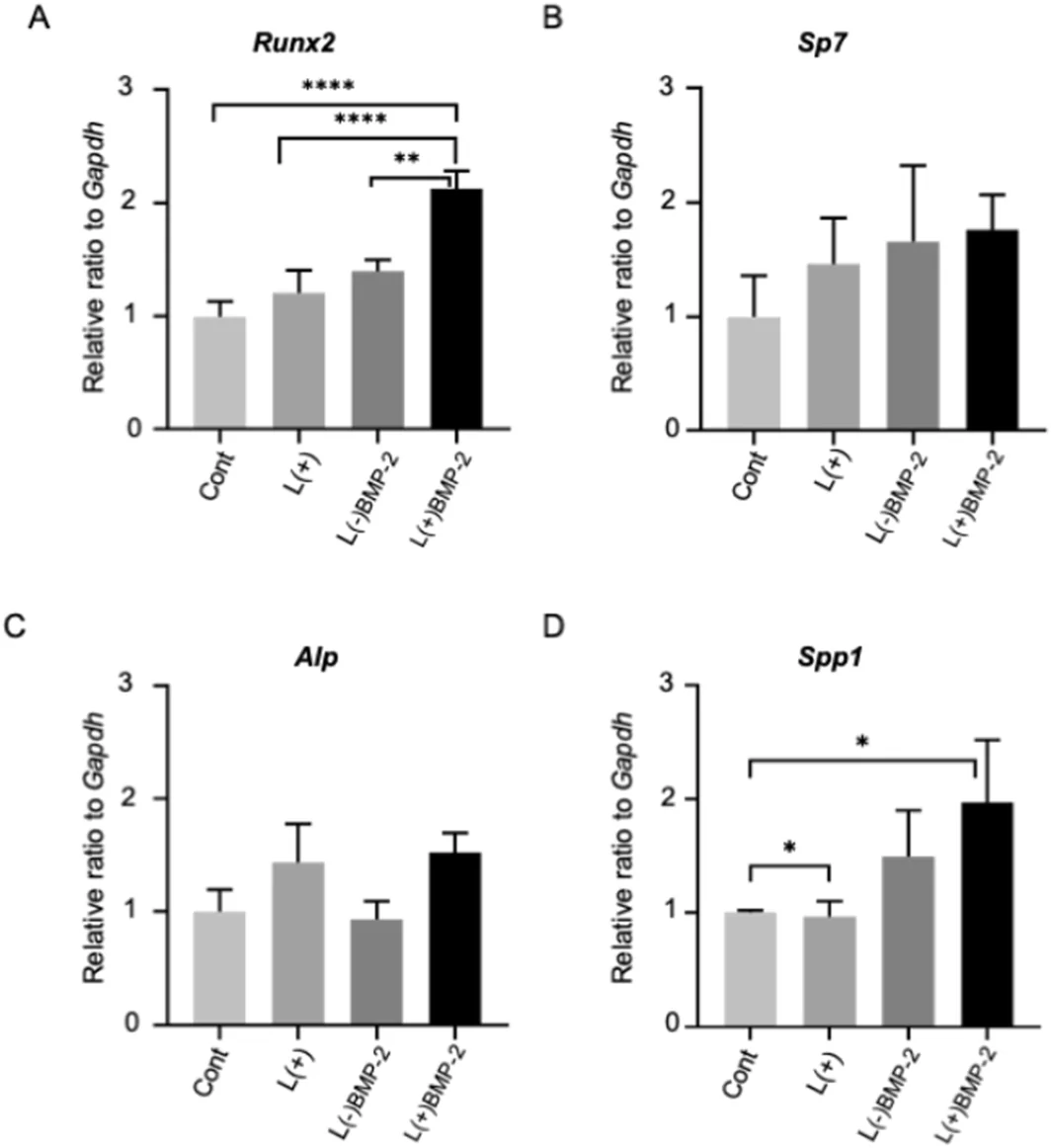
Combination of LIPUS treatment and BMP‐2 regulates osteogenic marker gene expressions. C3H10T1/2 cells were treated with BMP‐2 and/or LIPUS once per day during the final 3 days of the experiment. After 2 h of final treatment, total RNA was isolated and quantitative RT‐PCR analysis was performed. The amount of *Gapdh* mRNA was used as a standard. Delta–delta Ct (ΔΔCt) method was used for the quantification of the gene expression levels of *Runx2* (A), *Sp7* (B), *Alp* (C), *and Spp1* (D) with (*n* = 6) or without BMP‐2 and/or LIPUS (*n* = 6). The graphs represent mean ± SD with one‐way ANOVA Tukey's HSD post hoc test. (*p < 0.05, **p < 0.01, ***p < 0.001, and ****p < 0.0001). Cont: without BMP‐2 and LIPUS treatment; L (+): LIPUS treatment alone; L (−)BMP‐2: BMP‐2 stimulation alone; and L (+) BMP‐2: both BMP‐2 and LIPUS treatment.

Next, we evaluated the synergistical effect of LIPUS combined with BMP‐2 on osteogenic gene expressions, such as *Runx2*, *Sp7*, *Alp*, and *Spp1*, in C3H10T1/2 cells. As shown in Figure 2A, *Runx2*, which is a master gene of early osteoblast differentiation, was significantly upregulated by LIPUS combined with BMP‐2 compared with any other treatment group. Moreover, the gene expression of *Spp1*, which encodes osteopontin, also increased upon treatment with both LIPUS and BMP‐2 (Figure 2D). On the other hand, although the gene expressions of *Sp7* and *Alp* showed the trend to be upregulated by LIPUS combined with BMP‐2, compared to those with no stimulation, the effects had no significant differences (Figure 2B,C). These findings suggest that treatment with LIPUS or BMP‐2 alone was insufficient to commit the cells to osteogenic differentiation; however, treatment with the combination of LIPUS and BMP‐2 showed a strong ability to commit them to differentiate to osteoblasts.

### 3.3 Combination of LIPUS and BMP‐2 effects changes in the gene expression of CCN family members

**FIGURE 3:**
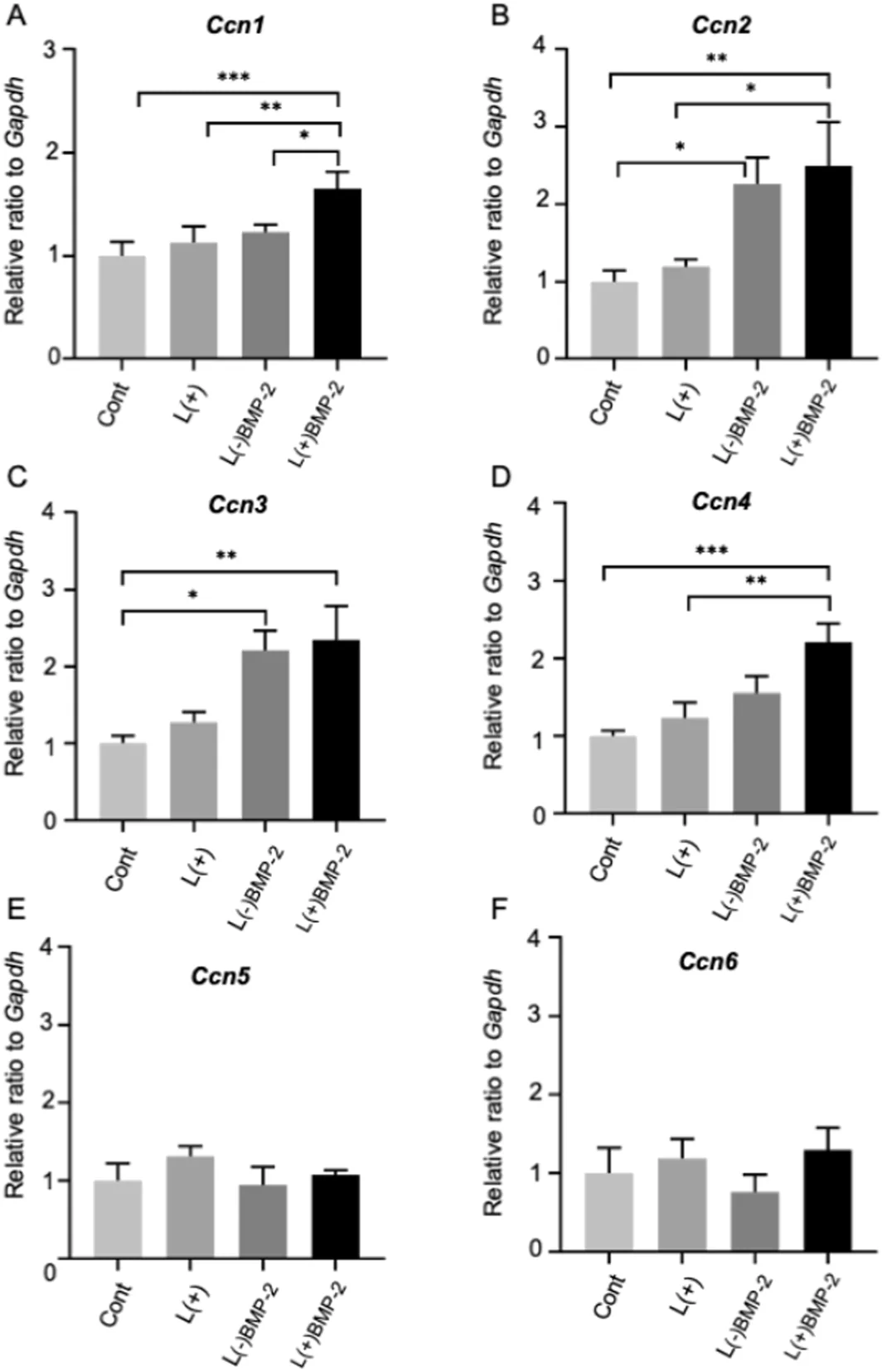
Combination of LIPUS treatment and BMP‐2 regulates the gene expressions of CCN family proteins. Experiments were performed under the same conditions as described in the Figure 2 caption. The gene expression levels of CCN family genes were examined by quantitative RT‐PCR analysis. The amounts of CCN family genes were normalized to the amount of *Gapdh* mRNA and were quantified by using the ΔΔCt method. The gene expressions of *Ccn1* (A), *Ccn2* (B), *Ccn3* (C), and *Ccn4* (D) were significantly increased by treatment with LIPUS combined with BMP‐2 compared with the control group. However, no effect was observed in either *Ccn5* (E) or *Ccn6* (F) expressions. The graphs represent mean ± SD with one‐way ANOVA Tukey's HSD post hoc test. (*n* = 6, *p < 0.05, **p < 0.01, and ***p < 0.001). Cont: without BMP‐2 and LIPUS treatment; L (+): LIPUS treatment alone; L (−)BMP‐2: BMP‐2 stimulation alone; and L (+) BMP‐2: both BMP‐2 and LIPUS treatment.

**FIGURE 4:**
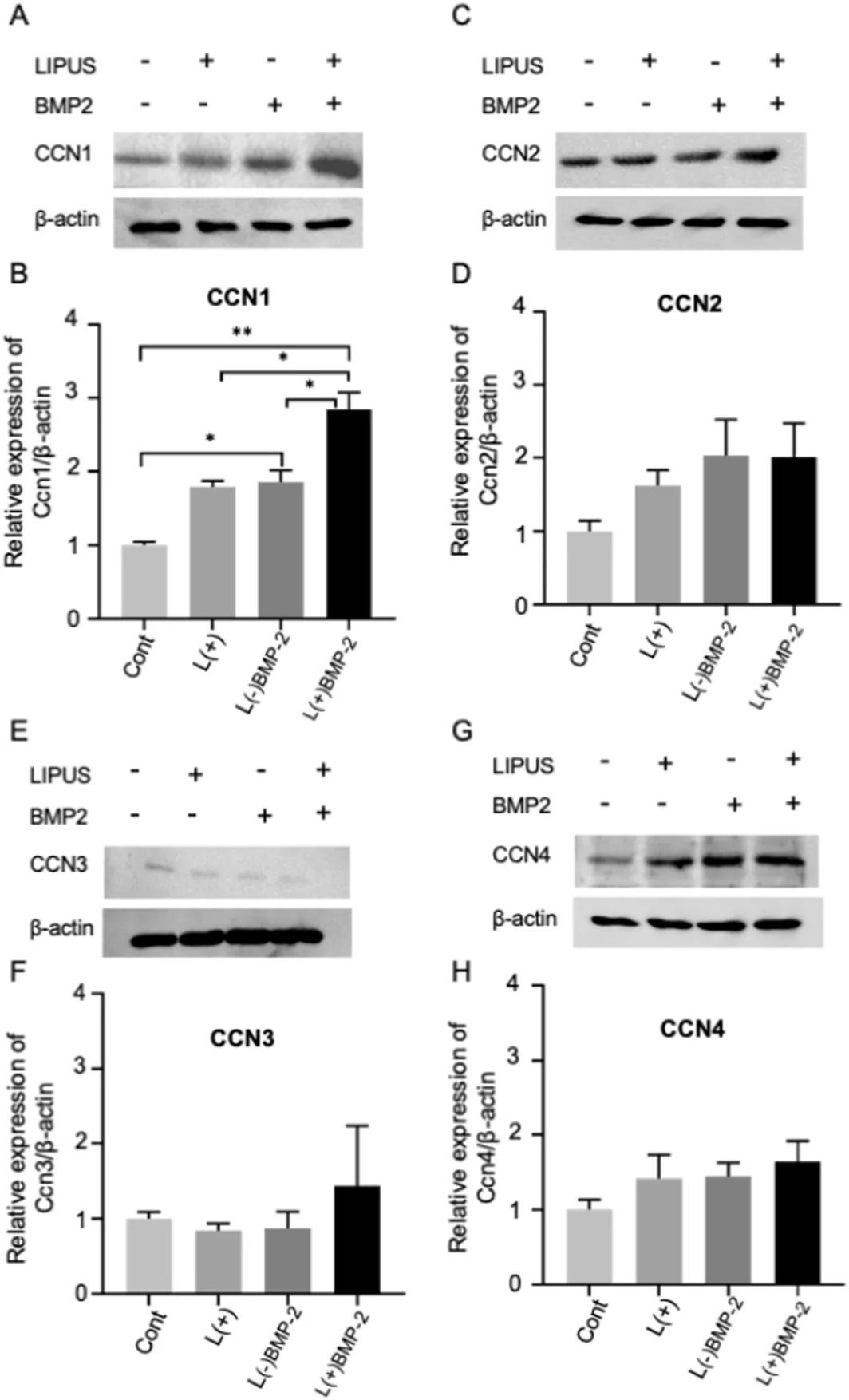
Production of CCN1, CCN2, CCN3, and CCN4 by treatment with combined LIPUS treatment with BMP‐2. Experimental conditions were the same as described in the Figure 2 caption. After 2 hours of stimulation on the final day with LIPUS and/or BMP‐2, the cell lysates were collected and western blot analysis was performed to investigate the protein productions of CCN1 (A), CCN2 (C), CCN3 (E), and CCN4 (G), which were significantly upregulated by the combination of LIPUS and BMP‐2 at mRNA level (Figure 3). As a loading control, the production of *β*‐actin was also analyzed (B, D, F, and H). The amounts of CCN1 (B), CCN2 (D), CCN3 (F), and CCN4 (H) were determined densitometrically using ImageJ and these amounts were normalized to the amount of *β*‐actin. Relative densities (Cont. = 1.0) from three independent experiments are presented, and the graphs represent mean ± SD with one‐way ANOVA Tukey's HSD post hoc test. (*p < 0.05 and **p < 0.01). Cont: without BMP‐2 and LIPUS treatment; L (+): LIPUS treatment alone; L (−)BMP‐2: BMP‐2 stimulation alone; and L (+) BMP‐2: both BMP‐2 and LIPUS treatment.

We previously reported that LIPUS‐induced CCN2 production suppressed adipogenesis under conditions committed to adipocyte differentiation.^11^ Based on this finding, we considered that the upregulation of CCN family members by LIPUS treatment may also be involved in the promotion of early osteogenic differentiation by LIPUS combined with BMP‐2. Hence, we next systematically examined CCN family gene expressions. As shown in Figure 3A–D, *Ccn1*, *Ccn2*, *Ccn3*, and *Ccn4* expressions exhibited the highest levels following treatment with the combination of LIPUS and BMP‐2, and those gene expressions showed significant differences against the controls. In contrast, *Ccn5* and *Ccn6* expressions showed no changes with any treatment (Figure 3E,F). To support these results, we investigated the protein productions of CCN1, CCN2, CCN3, and CCN4, which showed significant differences at gene expression levels upon western blot analysis. Although both CCN2 and CCN4 productions tended to be increased by combined treatment with LIPUS and BMP‐2, their enhancement had no statistical significance (Figure 4C,D,G,H). Additionally, the bands of CCN3 were faint, even if the *Ccn3* expression was upregulated (Figure 4E,F). On the other hand, CCN1 production was significantly increased by treatment with LIPUS combined with BMP‐2 compared with that in any other treatment group (Figure 4A,B). Collectively, these results indicate that the combination of LIPUS and BMP‐2 enhances both the gene expression and protein production of CCN1, thus suggesting that CCN1 may be involved in the regulation of *Runx2* expression, which plays an important role in early osteogenic differentiation.

### 3.4 Treatment with recombinant human CCN1 upregulates osteogenic marker gene expressions in C3H10T1/2 cells

**FIGURE 5:**
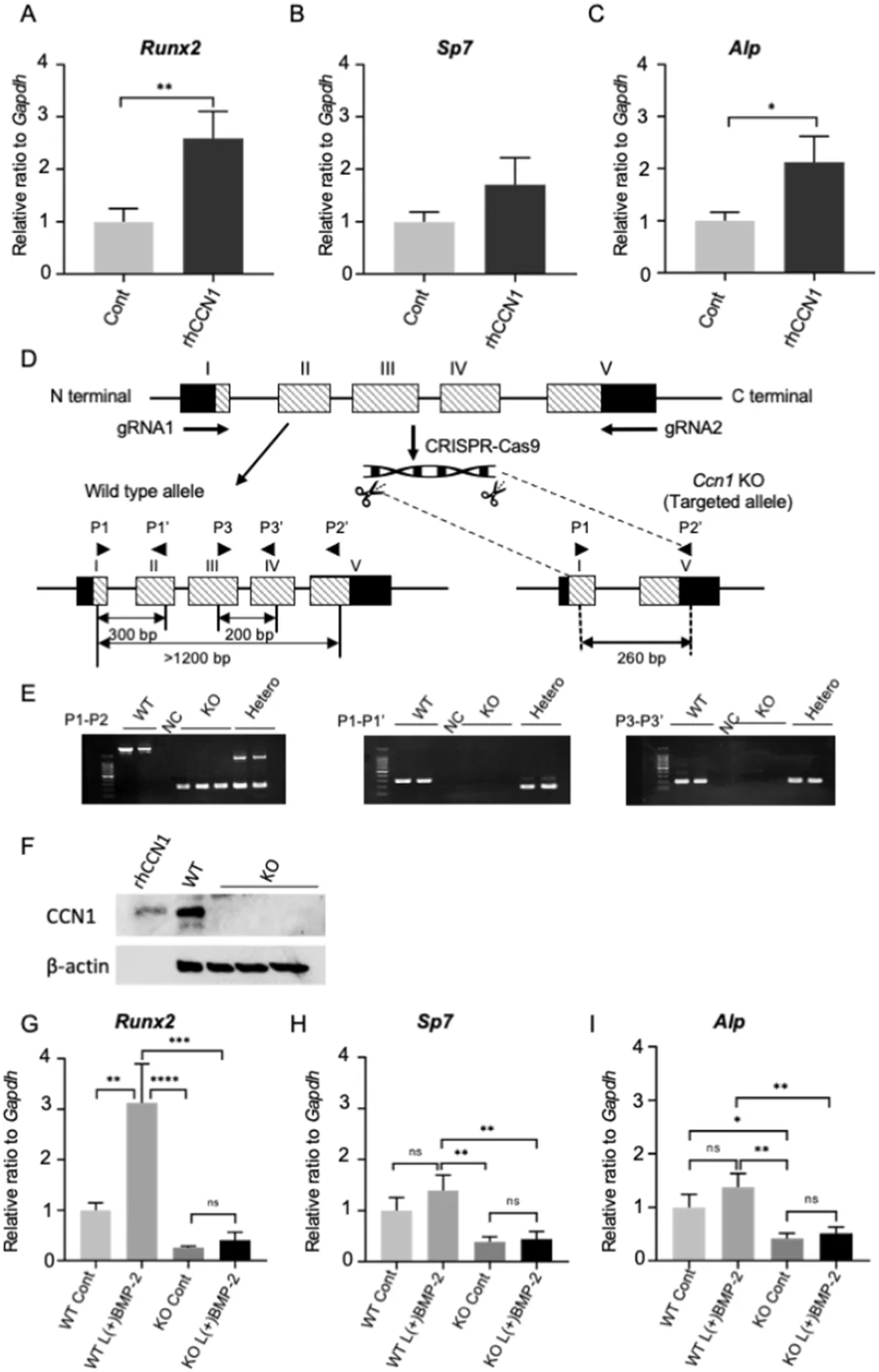
Effects of CCN1 on the gene expressions of osteogenic differentiation markers. C3H10T1/2 cells were cultured in osteogenic media, and then the cells were treated once with 100 ng/mL recombinant human CCN1 (rhCCN1) for 3 days, instead of the treatment with LIPUS combined with BMP‐2. Thereafter, quantitative RT‐PCR analysis was performed. The amount of *Gapdh* mRNA was used as a control and the gene expression levels of *Runx2* (A), *Sp7* (B), and *Alp* (C) were quantified after the treatment with vehicle (*n* = 6) or rhCCN1 (*n* = 6) using the ΔΔCt method. The ordinate indicates the relative ratio with respect to the vehicle treatment (Cont. = 1.0). The graphs represent mean ± SD with Student's *t* test results (**p < 0.01). Cont: treatment with vehicle and rhCCN1: treatment with rhCCN1. (D) Generation of *Ccn1* knockout C3H10T1/2 cells by CRISPR‐Cas9 technique. Schema shows the location of 2 guide RNAs targeting the N‐terminal and C‐terminal regions (arrows) of mouse *Ccn1*. The black and diagonally striped boxes indicate the noncoding exon and coding exon, which are indicated as I‐V of the *Ccn1* gene, respectively. Each arrowhead shows the primer binding site to detect gene deletion. The left and right panels indicate wild‐type (WT) and knockout alleles, respectively. (E) Left panel shows agarose gel electrophoresis of polymerase chain reaction (PCR) products of >1200 base pairs (bp) from WT cells (2 clones) and 260 bp from KO cells (3 clones) with primers 1 and 2’. Heterozygous allele revealed 2 bands of >1200 bp and 260 bp. In middle and right panels, both WT and heterozygous allele showed bands of 300 bp or 200 bp by PCR analysis with primer 1 and 1′ or primer 3 and 3′, respectively, whereas KO allele did not. As a negative control, distilled H_2_O without genomic DNA was used. (F) Western blot analysis was performed to confirm the loss of CCN1 protein in *Ccn1* KO cells. rhCCN1 was used as a positive control. As a loading control, production of *β*‐actin was also analyzed. (G–I) The gene expressions of *Runx2* (G), *Sp7* (H), and *Alp* (I) were examined in WT and *Ccn1* KO cells treated with LIPUS combined with BMP‐2. The amounts of these genes were not upregulated in KO cells, even if LIPUS and BMP‐2 were applied. The graphs represent mean ± SD with one‐way ANOVA Tukey's HSD post hoc test results (*n* = 6, *p < 0.05, **p < 0.01, ***p < 0.001, ****p < 0.0001, and ns: not significant). WT Cont: WT cells without BMP‐2 and LIPUS treatment; WT L (+) BMP‐2: WT cells treated with LIPUS treatment and BMP‐2; KO Cont: KO cells without BMP‐2 and LIPUS treatment; and KO L (+) BMP‐2: WT cells treated with LIPUS treatment and BMP‐2.

Since CCN1 was strongly induced by treatment with LIPUS combined with BMP‐2, we hypothesized that CCN1 may be the main regulator of osteogenic gene expressions. Hence, we investigated whether or not the gene expressions of osteogenic markers were upregulated by treatment with rhCCN1 alone, instead of treatment with LIPUS combined with BMP‐2. As shown in Figure 5A,C, *Runx2* and *Alp* expressions were significantly upregulated in rhCCN1‐treated cells compared with the vehicle‐treated cells. On the other hand, although *Sp7* expression level did not reach statistical significance, it showed an increasing trend with rhCCN1 (Figure 5B). Taken together, these results demonstrate that CCN1 alone is sufficient to promote the gene expressions of *Runx2* and *Alp*, which encode the key factors for osteogenic differentiation in MSCs.

### 3.5 Generation of Ccn1‐deficient C3H10T1/2 cells and the effect of Ccn1 deficiency on osteogenic marker gene expressions

To further elucidate the functional roles of CCN1 during osteogenic differentiation, we generated *Ccn1*‐deficient C3H10T1/2 cells (*Ccn1*‐KO cells) using a CRISPR–Cas9 technique. Two gRNAs (gRNA1 and gRNA2) targeting the Exon I and Exon V of the mouse *Ccn1* gene were designed to induce a deletion of the complete *Ccn1* coding region (Figure 5D bold arrows). Genomic PCR analysis using primer set P1–P2′, which detects the full length of the *Ccn1* coding sequence, revealed that amplified PCR products of wild‐type homozygote resulted in >1200 bp and those of *Ccn1*‐KO homozygote showed the expected 260 bp fragment (Figure 5E, left panel). The heterozygote showed two bands (Figure 5E, left panel). Moreover, genomic PCR analyses using primer sets P1‐P1′ and P3‐P3′ were performed to detect the N‐terminus coding region and a segment between Exon III and Exon IV of *Ccn1*, respectively. As expected, no PCR products from the genome of *Ccn1*‐KO cells were amplified (Figure 5E, middle and right panels). Furthermore, consistent with the results of genomic PCR analysis, CCN1 production was not detected in *Ccn1*‐KO cells by western blot analysis, whereas CCN1 production was clearly detected in wild‐type cells (Figure 5F). These results indicate that the generation of *Ccn1*‐deficient C3H10T1/2 cells was successful. Next, using *Ccn1*‐KO cells, we examined the effects of LIPUS combined with BMP‐2 on the expressions of osteogenic differentiation markers. As shown in Figure 5, panels G to I, when wild‐type and *Ccn1*‐KO cells were treated with LIPUS combined with BMP‐2, *Runx2*, *Sp7*, and *Alp*, expressions in *Ccn1*‐KO cells were significantly decreased compared with those in cells. On the other hand, in wild‐type cells treated with the combination, although *Sp7* and *Alp* expressions showed a trend of increasing without statistical significance (Figure 5H,I), *Runx2* expression was significantly increased, similar to the results in Figure 2 (Figure 5G). Finally, LIPUS combined with BMP‐2 had no effect on all gene expressions in *Ccn1*‐KO cells (Figure 5 panels G to I). These results suggest that CCN1, which is produced by the combination, contributes to the upregulation of *Runx2* expression during osteogenic differentiation.

### 3.6 Runx2 expression decreased by Ccn1‐deficiency is rescued by the forced expression of Ccn1

**FIGURE 6:**
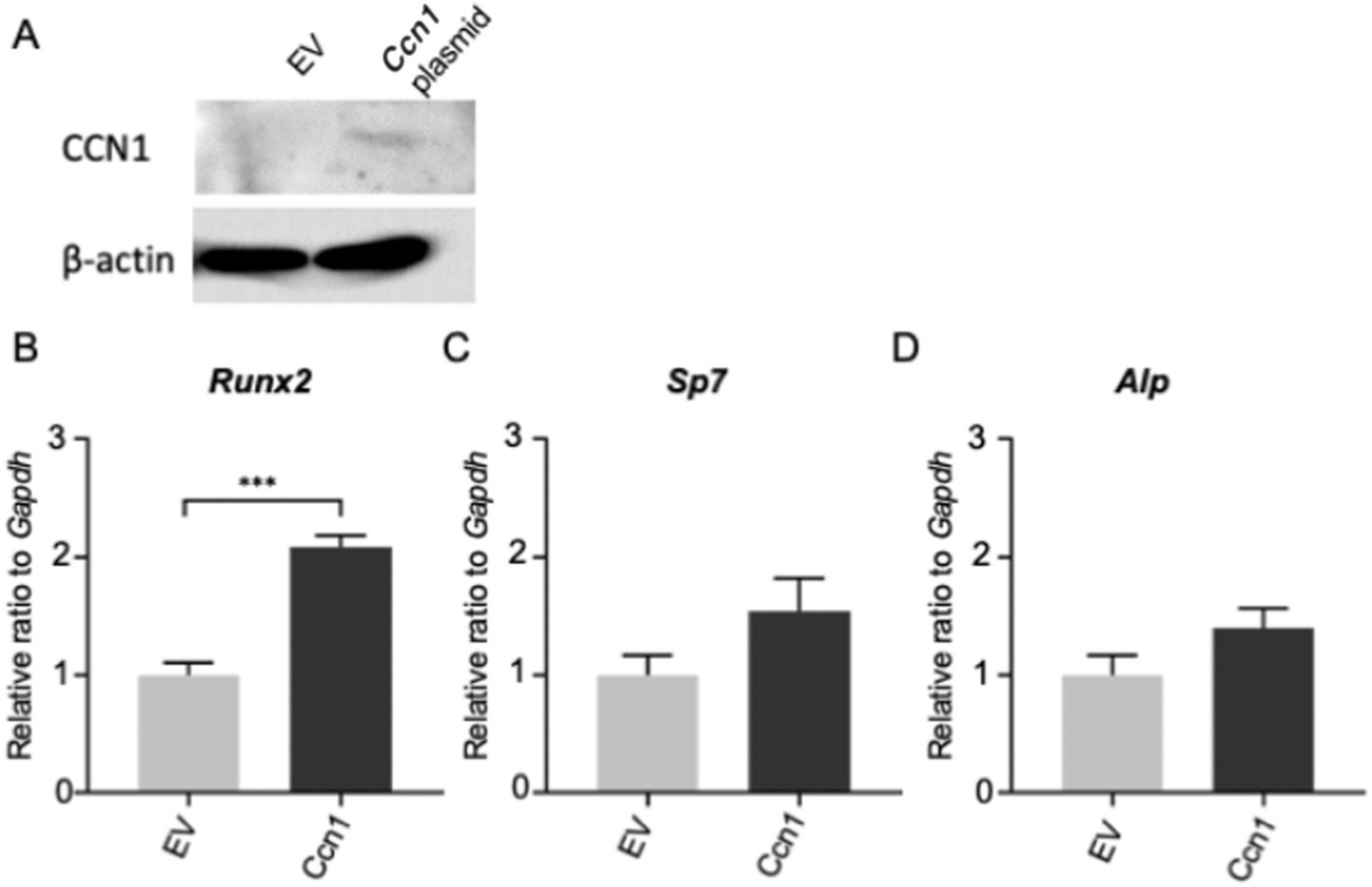
Restoration of osteogenic marker genes in *Ccn1* KO cells transfected with *Ccn1* expression plasmid. To confirm the rescue by the forced expression of CCN1, *Ccn1*‐KO cells were transfected with either the *Ccn1* expression plasmid or empty vector (EV) and cultured under standard conditions. Cell lysate and total RNAs were collected 48 h after transfection. (A) Western blot analysis was performed to confirm the production of CCN1 in *Ccn1* KO cells. Although CCN1 production was not detected by transfection with EV, the CCN1 band was detected by transfection with the *Ccn1* expression vector. (B–D) The gene expression levels of *Runx2* (B), *Sp7* (C), and *Alp* (D) were evaluated in *Ccn1* KO cells transfected with either EV or the *Ccn1* expression plasmid. The amount of *Runx2* mRNA was redeemed by the forced expression of CCN1. Data represent mean ± SD with Student's *t* test (*n* = 6 and ***p < 0.001).

To further confirm that CCN1 plays an important role in *Runx2* expression, we investigated whether or not the decreased gene expressions of osteogenic markers are rescued in *Ccn1*‐KO cells by transfection with a *Ccn1* expression plasmid. Firstly, we confirmed that CCN1 was reproduced in *Ccn1*‐KO cells transfected with a *Ccn1* expression plasmid by western blot analysis (Figure 6A). Then, we investigated the expressions of osteogenic marker genes. As shown in Figure 6B, *Runx2* expression impaired by *Ccn1* deficiency was significantly increased in *Ccn1*‐transfected cells compared with that in EV‐transfected cells. Moreover, the expression levels of *Sp7* and *Alp* trended toward elevation in *Ccn1*‐transfected cells, although these gene expression levels had no statistical significance (Figure 6C,D). These findings indicate that the forced expression of CCN1 restores *Runx2* expression, confirming that CCN1 works as a crucial factor for the regulation of early osteogenic differentiation by LIPUS combined with BMP‐2.

## 4 DISCUSSION

This study demonstrated that the gene expression of *Runx2*, which is a master transcription factor for early osteoblast differentiation, was upregulated by LIPUS combined with BMP‐2 through CCN1 production. As one of the mechanisms of this action, we showed that treatment with their combination enhanced the gene expressions of BMP receptors and markedly activated Smad1/5/9, which constitutes the downstream of the canonical BMP signaling pathway. These findings suggest that the biological activity of BMP‐2 is synergically enhanced by mechanical stimulation, such as LIPUS. So far, it has been reported that mechanical stress regulates the polymerization of actin fibers and subsequently determines the direction of MSC differentiation through the nuclear translocation of yes‐associated protein 1 (YAP1)/TAZ.^24^ Since CCN1 and CCN2 have been known as the target genes of YAP1/TAZ,^25^, ^26^ the nuclear translocation of YAP1/TAZ by mechanical stress is considered to lead to upregulation of CCN1 and CCN2. In fact, our previous data revealed that CCN2 expression was significantly increased in adipocyte‐committed C3H10T1/2 cells and 3T3‐L1 cells treated with LIPUS,^12^ and as a result, adipogenesis was suppressed. Moreover, other reports showed that CCN1 production was promoted by mechanical loading.^27^ Reciprocal inhibitory relationships between adipogenesis and osteogenesis,^8^ between adipogenesis and myogenesis,^9^ or between myogenesis and osteogenesis^28^ have been found in the differentiation of MSCs. However, the treatment of osteogenesis‐committed C3H10T1/2 cells with LIPUS that suppressed adipogenesis had no effect on the gene expressions of *Runx2*, *Sp7*, and *Alp*. Based on these results, we hypothesized that the suppression of adipogenesis by mechanical stress does not sufficiently lead to osteogenesis per se, but rather requires another osteoinductive factor for osteoblast differentiation. Therefore, we used BMP‐2 as an osteoinductive factor, and we combined it with LIPUS. As expected, the gene expression of *Runx2*, which is a marker of early osteogenesis, was significantly upregulated. However, the gene expressions of *Sp7* and *Alp*, which are markers of late osteogenesis, trended to increase, but had no statistical significance. These results indicate that although early osteogenesis is promoted by both mechanical stress, such as LIPUS, and stimulation by BMP‐2, subsequent osteoblast differentiation may require stimulation with continuous BMP‐2 or another factor.

This study showed that the gene expressions of CCN1, CCN2, and CCN4, which are reported to promote osteogenesis among CCN family proteins,^17^ were significantly upregulated by LIPUS combined with BMP‐2. However, at the protein level, the production of only CCN1 was markedly increased. Therefore, we focused on the role of CCN1 in early osteogenic differentiation. CCN1 is a matricellular protein belonging to the CCN family of proteins, which are characterized by having four conserved module structures.^17^, ^29^, ^30^ It has been reported that CCN1 regulates not only cell survival,^31^ embryogenesis,^32^ wound healing,^33^ tumorigenesis,^32^ and cellular senescence^34^ but also tissue formation, through such as angiogenesis,^35^ myogenesis,^36^ and osteogenesis^37^ from MSCs. Additionally, a recent study revealed that CCN1 accumulated more in extracellular matrices (ECMs) produced by stromal cells derived from young (<25 years old) human donors than from older (>65 years old) donors.^38^ Moreover, when MSCs treated with BMP‐2 were cultured on ECMs derived from young donors, the expression of *Runx2* was significantly increased, but that of PPARγ, a master transcription factor for adipogenesis, was not.^38^ These results indicate that CCN1 increases the expression level of *Runx2* in the presence of BMP‐2. Furthermore, it has also been reported that CCN1 interacts with integrin αvβ3 and increases BMP‐2 expression.^39^ In addition, we have found that combined treatment of LIPUS and BMP‐2 markedly enhanced the phosphorylation of ERK1/2, suggesting the activation of MAPK signaling pathways downstream of integrin‐mediated mechanotransduction. These findings suggest that ERK1/2 activation may contribute to the regulation of *Runx2* expression during early osteogenic differentiation. Taken together with our previous report that the phosphorylation of ERK1/2 is induced by LIPUS treatment in chondrocytes,^20^ it is indicated that CCN1 is produced from MSCs by simultaneous stimulation by LIPUS and BMP‐2 and interacts with integrins to promote the phosphorylation of ERK1/2 downstream of the integrin signaling. As a result, CCN1 increases the *Runx2* expression and promotes early osteogenic differentiation of MSCs. Furthermore, we previously reported that CCN2 interacted with BMP‐2 and that their interaction promoted chondrocyte differentiation more than BMP‐2 alone.^40^ Considering that CCN1 is structurally and functionally similar to CCN2, it is presumed that CCN1 may also interact with BMP‐2, and CCN1‐complexed BMP‐2 may possibly enhance BMP signaling more. Indeed, we showed that the phosphorylation of Smad1/5/9 was remarkably increased by the combination of LIPUS and BMP‐2. Considering that CCN1 production is enhanced by treatment with LIPUS and BMP‐2, these data support the presumption that BMP signaling may be enhanced by the interaction of CCN1 and BMP‐2. Collectively, when MSCs are treated with combined LIPUS and BMP‐2, MSC‐produced CCN1 interacts with BMP‐2 in ECM, and their complex binds to BMP receptors and strongly enhances BMP signaling. As a result, enhanced BMP signaling upregulates the *Runx2* expression, resulting in the promotion of early osteogenesis of MSCs. However, further investigation is needed to clarify this mechanism of promotion of early osteogenesis by combined LIPUS and BMP‐2.

The early achievement of osseointegration by fixing the dental implant directly into the alveolar bone is important for successful dental implant therapy.^1^ Therefore, thus far, LIPUS treatment has often been clinically performed to achieve early osseointegration.^41^ The results of this study demonstrate that LIPUS treatment in the presence of BMP‐2 promotes the early osteogenesis of MSCs through the enhancement of CCN1 production, thus suggesting that LIPUS combined with BMP‐2 or the application of CCN1 is a novel strategy for successful osseointegration.

## AUTHOR CONTRIBUTIONS

**Hsu Myat Paing**: Writing—original draft; investigation; formal analysis; and data curation. **Satoshi Kubota**: Writing—review and editing; supervision; funding acquisition; validation; and project administration. **Masaharu Takigawa**: Writing—review and editing; funding acquisition; validation. **Takuo Kuboki**: writing—review and editing; validation; project administration. **Takashi Nishida**: Writing—original draft; supervision; investigation; data curation; formal analysis; validation; project administration; methodology; visualization; funding acquisition; conceptualization.

## CONFLICT OF INTEREST STATEMENT

M.T. obtained research funding from ITO Co., Ltd. All other authors declare no conflicts of interest.

## ETHICS STATEMENT

This study did not require any ethics approval.

## Supporting information

Supporting Information S1

Supporting Information S2

Figure S1

Figure S2

Table S1

Table S2

## Data Availability

The data that support the findings of this study are available from the corresponding author upon reasonable request.
