# Supplementary material for: Critical role of cellular communication network factor 1 in early osteogenesis of mesenchymal stem cells primed by low‐intensity pulsed ultrasound and bone morphogenetic protein 2
Source: J Cell Commun Signal. 2026 Aug 26;20(3):e70101. doi: 10.1002/ccs3.70101 (PMC13513212; doi:10.1002/ccs3.70101)
Supplement: Supplementary file 1 [file CCS3-20-e70101-s002.docx]

**Supplementary Fig. S1 caption**

**Effect of LIPUS treatment on the expressions of osteogenic marker genes in adipocyte-committed C3H10T1/2 cells.**

C3H10T1/2 cells were inoculated at a density of 1.0 x 10^5^ cells into 35 mm culture dishes, and the next day the cells were differentiated to adipocytes according to the protocol as previously indicated [12]. LIPUS treatment was performed 4 times at a frequency of once a day during the 4-day experimental periods. After 4 h of the last treatment with LIPUS, total RNAs were isolated and a quantitative RT-PCR analysis was performed. The amount of *Gapdh* mRNA was used as a control. The delta-delta Ct method was used for the relative quantification of the expressions of osteogenic marker genes, such as *Runx2* (A), *Sp7* (B), and *Alp* (C). The bars represent mean and standard deviation data from independent culture dishes (n = 5).
