# Supplementary material for: Critical role of cellular communication network factor 1 in early osteogenesis of mesenchymal stem cells primed by low‐intensity pulsed ultrasound and bone morphogenetic protein 2
Source: J Cell Commun Signal. 2026 Aug 26;20(3):e70101. doi: 10.1002/ccs3.70101 (PMC13513212; doi:10.1002/ccs3.70101)
Supplement: Supplementary file 2 [file CCS3-20-e70101-s003.docx]

**Supplementary Table 1**

| REAGENT or RESOURCE | SOURCE | IDENTIFIER |
| --- | --- | --- |
| Antibodies | | |
| Rabbit polyclonal anti-CCN1 | Proteintech | Cat#26689-1-AP; RRID: AB_2880604 |
| Rabbit polyclonal anti-CCN2 | Proteintech | Cat#25474-1-AP; RRID: AB_2918089 |
| Rabbit polyclonal anti-CCN3 | Abcam | Cat#ab10888;  RRID: AB_301511 |
| Rabbit polyclonal anti-CCN4 | Santa Cruz Biotechnology | Cat#sc-25441;  RRID: AB_2214691 |
| Rabbit monoclonal anti-phospho-Smad1/5/9 | Cell Signaling Technology | Cat#13820S;  RRID: AB_2491015 |
| Rabbit monoclonal anti-phospho-ERK1/2 | Cell Signaling Technology | Cat#9106S;  RRID: AB_331768 |
| Rabbit monoclonal anti-ERK1/2 | Cell Signaling Technology | Cat#9102;  RRID: AB_330744 |
| HRP-conjugated anti-rabbit IgG | Cell Signaling Technology | Cat#7074S;  RRID: AB_2099233 |
| HRP-conjugated anti-mouse IgG | Cell Signaling Technology | Cat#7076S;  RRID: AB_330924 |
| Mouse monoclonal anti-β-actin | MilliporeSigma | Cat#A2228;  RRID: AB_476697 |
| Chemicals, peptides, and recombinant proteins | | |
| Recombinant BMP-2 protein | Osteopharma Inc. | N/A |
| Recombinant CCN1 protein | PeproTech | Cat# 120-25 |
| Recombinant Cas9 protein | Takara Bio | Cat# 632641 |
| Disodium β-glycerophosphate pentahydrate | MilliporeSigma | Cat#28-2840; |
| Ascorbic acid | Nacalai Tesque | Cat#13570-66 |
| Critical commercial assays | | |
| RNeasy Mini Kit | Qiagen | Cat#74106 |
| PrimeScript RT Reagent Kit | Takara Bio | Cat#RR037A |
| SYBR Green Real-time PCR Master Mix | Toyobo | Cat# QPK-201 |
| BCA Protein Assay Kit | Thermo Fisher Scientific | Cat# 23225 |
| GeneArt Precision gRNA Synthesis Kit | Invitrogen | Cat# A29377 |
| Quick Taq HS DyeMix | Toyobo | Cat# DTM-101 |
| Experimental models: Cell lines | | |
| The murine mesenchymal stem cell line  C3H10T1/2 | RIKEN Cell Bank | RCB0247  RRID: CVCL_0190 |
| Recombinant DNA | | |
| pmaxGFP plasmid | Lonza | Cat# V014-332 |
| *Ccn1* expression plasmid (pFlag-CMV) | This paper | N/A |
| pFlag-CMV empty vector | Invitrogen | N/A |
| Other | | |
| ST-SONIC ultrasound apparatus | ITO Corporation | Model: ST- SONIC |
| StepOnePlus Real-Time PCR System | Applied Biosystems | Cat# 4376600; |
| DU 730 spectrophotometer | Beckman Coulter | Cat# A23616 |
| NEPA21 electroporator | NEPA Gene | NEPA21 TypeII |
| Semi-dry transfer apparatus | Atto | N/A |
| LAS-4000 imaging system | Fujifilm | LS-4000 mini |
