# Supplementary material for: Critical role of cellular communication network factor 1 in early osteogenesis of mesenchymal stem cells primed by low‐intensity pulsed ultrasound and bone morphogenetic protein 2
Source: J Cell Commun Signal. 2026 Aug 26;20(3):e70101. doi: 10.1002/ccs3.70101 (PMC13513212; doi:10.1002/ccs3.70101)
Supplement: Supplementary file 3 [file CCS3-20-e70101-s004.docx]

**Supplementary Fig. S2.**

**Transfection efficiency of *Ccn1* expression plasmid into *Ccn1*-KO cells.**

To confirm the efficient transfection of the *Ccn1* expression plasmid, pmaxGFP plasmid was also co-transfected with the *Ccn1* expression plasmid or empty vector (EV) into *Ccn1*-KO cells. After 48 h, GFP-positive cells were observed under fluorescence microscopy. Representative images of *Ccn1*-KO cells transfected with either EV plus pmaxGFP or the *Ccn1* expression plasmid plus pmaxGFP. Left-panel, Bright-field; middle panel, fluorescence of GFP; and right-panel, merged images. The bars represent 50 μm.
