# Supplementary material for: Critical role of cellular communication network factor 1 in early osteogenesis of mesenchymal stem cells primed by low‐intensity pulsed ultrasound and bone morphogenetic protein 2
Source: J Cell Commun Signal. 2026 Aug 26;20(3):e70101. doi: 10.1002/ccs3.70101 (PMC13513212; doi:10.1002/ccs3.70101)
Supplement: Supplementary file 4 [file CCS3-20-e70101-s005.docx]

**Supplementary Table 2.**

(A) Sequences of forward and reverse primers used for quantitative PCR.

| Gene | Forward | Reverse |
| --- | --- | --- |
| *Runx2* | TCGTCAGCATCCTATCAGT | CAGCGTCAACACCATCAT |
| *Sp7* | ACGATGATGATGATGATGATGATGAT | AACACCAATCTCCTTAACTCTG |
| *Alp* | GCTCTCCCTACGCACCCTGTTC | TGCTGGAAGTTGCCTGGACCTC |
| *Spp1* | ACACTTTCACTCCAATCGTCC | TGCCCTTTCCGTTGTTGTCC |
| *Bmpr2* | CAAATATCCTGGATGGCAGCAGTA | TGGAGATGACCCAGGTGGAC |
| *Bmpr1a* | ATGGCCATTGCTTTGCCATTA | TCCTGCGTAGCTGGGCTTTC |
| *Bmpr1b* | CTACGTTGTAAATGCCACCACCA | AGGTGACAACAGGCATTCCAGA |
| *Bmp2* | TGACTGGATCGTGGCACCTC | CAGAGTCTGCACTATGGCATGGTTA |
| *Ccn1* | ATGAAGACAGCATTAAGGACTC | TGCAGAGGGTTGAAAAGAAC |
| *Ccn2* | CCACCCCAGTTACCAATGAC | GTGCAGCCAGAAAGCTCA |
| *Ccn3* | TGAAGTCTCTGACTCCAGCATT | TGGCTTTCAGGGATTTCTTG |
| *Ccn4* | TGAGAACTGCATAGCCTACAC | TACACAGCCAGGCATTTC |
| *Ccn5* | GCTGTGATGACGGTGGTT | GACAAGGGCAGAAAGTTGG |
| *Ccn6* | GTGCAAAGTCTGTGCCAAG | GAACTCACATCCAACTGCCA |
| *Gapdh* | GCCAAAAGGGTCATCATCTC | GTCTTCTGGGTGGCAGTGAT |

(B) Sequences of forward and reverse primers used to create guide RNAs.

| Target region | Forward | Reverse |
| --- | --- | --- |
| N-terminal region | TAATACGACTCACTATGTGTGGCGCGCAGGAGGAACC | TTCTAGCTCTAAAACCCGGTTCCTCCTGCGCGCCACA |
| C-terminal target | TAATACGACTCACTATAGATGCGGTTCCGATGCGAAGA | TTCTAGCTCTAAAACTCTTCGCATCGGAACCGCAT |

(C) Sequences of forward and reverse primers used for genomic PCR.

| Primer | Forward | Reverse |
| --- | --- | --- |
| P1-P1’ | AGCACCTCGAGAGAAGGACAC | CAGTCTGGTCAAGTGGAGAAGG |
| P2’ |  | TTGTGGATGTCATTGAATAGGC |
| P3-P3’ | ATGAAGACAGCATTAAGGACTC | TGCAGAGGGTTGAAAAGAAC |

(D) Sequences of forward and reverse primers used to create *Ccn1* expression plasmid.

(restriction site: lower cases)

| Position | Forward | Reverse |
| --- | --- | --- |
| 188-1330 | cggaattcCGCCACAATGAGCTCCAGCACC | cgggatccCTA*ATGATGATGATGATGATGATGATG*GTCCCTGAACTTGTGGATGTC |

(8xHis-tag : Italic cases)
